# 62. A case of destructive arthritis with nodules

**DOI:** 10.1093/rap/rky034.025

**Published:** 2018-09-20

**Authors:** Babitha Mekkayil, Muddassir Shaikh, Michael Plant

**Affiliations:** Rheumatology, James Cook University Hospital, Ingleby Barwick, UNITED KINGDOM

**Introduction:** Multicentric reticulohistiocytosis (MRH) is a rare multisystem, granulomatous condition is an important differential diagnosis in a patient presenting with mutilating arthritis and multiple cutaneous nodules.

**Case description:** A 64 year old man presented with nine months history of pain, swelling and deformity of the small joints of the fingers and knees. Few months later he developed a dermal nodules distributed over the distal parts of the fingers. Inflammatory markers were normal: autoantibody screen was negative except for a significantly raised anti-ccp. Radiographs showed erosive arthropathy with normal bone density. The initial diagnosis was an erosive arthropathy, probably a seronegative inflammatory arthritis. He was commenced on prednisolone 30mg/day with good relief. Biopsy of the finger nodules showed mononuclear histiocytic cells and giant multinucleated cells occupying the entire thickness of the reticular and papillary dermis and a part of the subcutaneous fatty tissue .The clinical picture and the biopsy findings were consistent with multicentric reticulohistiocytosis which stained positively with CD68. He was treated with immunosuppressive therapy (cyclophosphamide and corticosteroids). His arthritis and dermal nodules responded well to treatment. Two years later the condition relapsed with new nodules on the upper limbs and face. Response to a further course of cyclophosphamide was less impressive. He was commenced on methotrexate which also hasn’t given good response and he is now under consideration for infliximab anti-TNF therapy.

**Discussion:** MRH although is a very care condition, it can be very destructive and needs appropriate treatment. This can be also associated with malignancy and needs thorough evaluation. Although there is no therapy that cures the condition, immunosuppressive medications play an active role. It has shown that after a course of 7-8 years, patients often go into remission, but considerable joint destruction may have already occurred. Joint replacement may improve function in patients with burned out disease. Although many different drugs were used in the condition, it is difficult to determine the efficacy due to the rarity of the disease, lack of controlled studies and tendency for the remission

**Key Learning Points:** Clinicians should be aware of multicentric reticulohistiocytosis in patients presenting with inflammatory arthritis and nodules given the destructive nature and association with malignancy.

**Disclosure: B. Mekkayil:** None. **M. Shaikh:** None. **M. Plant:** None.

